# The Growth Modulation Index (GMI) as an Efficacy Outcome in Cancer Clinical Trials: A Scoping Review with Suggested Reporting Guidelines

**DOI:** 10.1007/s11912-025-01667-1

**Published:** 2025-03-29

**Authors:** Kilian Trin, Cynthia Dalleau, Simone Mathoulin-Pelissier, Christophe Le Tourneau, Derek Dinart, Carine Bellera

**Affiliations:** 1 INSERM CIC-1401, Clinical and Epidemiological Research Unit, Institut Bergonié, Comprehensive Cancer Center, Bordeaux, France; 2 Medical Science Faculty, University of Bordeaux, Bordeaux, France; 3 ISPED, Centre INSERM U1219 Bordeaux Population Health, Epicene Team, University of Bordeaux, Bordeaux, France; 4 Department of Drug Development and Innovation (D3i), Institut Curie, Paris, France; 5 INSERM U900 Research Unit, Institut Curie, Paris, France; 6 Paris-Saclay University, Paris, France

**Keywords:** Growth modulation index, Progression-free survival ratio, Time to progression ratio, Scoping review, Oncology, Guidelines

## Abstract

**Purpose of Review:**

The growth modulation index (GMI) is defined as the ratio between the time to progression of a new line of treatment and the previous line. This ratio can be used to determine whether the new line of treatment brings a clinical benefit. It has been proposed as an outcome in trials evaluating non-cytotoxic drugs. Its interest lies in the intra-patient comparison. The terminology employed to refer to the GMI, as well as its definitions, are highly variable in the literature. Some uses of the GMI are arbitrary and not based on any scientific rationale. Our aim is to describe how the GMI is reported in the scientific literature.

**Recent Findings:**

We carried out a scoping review using PubMed, Scopus, Web of Science and BASE (Bielefeld Academic Search Engine). The algorithm was composed of the terms "growth modulation index", "time to progression ratio" and "progression-free survival ratio". Documents in English, with full-text available, published up to 2023, were included. Among 227 included documents, 166 of which discussed GMI specifically. On these 166 documents, 76 reported on observational studies, 62 on interventional studies and 17 on methodological or statistical developments pertaining to the GMI. All were about oncology.

**Summary:**

Our review highlights significant variability in the reporting and use of the GMI. To address this, we propose standardized reporting guidelines. Additionally, we emphasize the need for methodological and statistical developments to improve the use of the GMI and to develop novel GMI-based trial designs.

**Supplementary Information:**

The online version contains supplementary material available at 10.1007/s11912-025-01667-1.

## Introduction

In confirmatory cancer trials, overall survival and quality of life are the gold standards to establish efficacy of a new treatment. Intermediate outcomes such as tumour shrinkage are commonly used in earlier phases of clinical trial development. In the context of early phase trials evaluating non-cytotoxic drugs, an outcome accounting for the time to progression may be more appropriate than simply measuring tumour shrinkage [1, 2]. The growth modulation index (GMI) was defined originally as the ratio of the time to progression (TTP) of a new treatment line (TTP2) to the TTP of the previous treatment line (TTP1) [1]. This ratio can be used to determine whether current therapy provides clinical benefit as compared to the previous one. It was proposed as a novel efficacy outcome for the evaluation of non-cytotoxic drugs. Because of the natural history of the disease, one would expect that TTP2 be shorter than TTP1, drug efficacy being equal, which would indicate a ratio value smaller than 1. A GMI superior to 1 would thus indicate efficacy of the second-line treatment [3, 4]. Considering that targeted treatment may generate significant clinical benefits aside from tumour shrinkage, GMI has since been applied in early drug development settings to assess the benefit of targeted therapies selected by molecular profiling in patients with advanced refractory cancers [5]. The benefit of using GMI is that patients act as their own controls, allowing the direct comparison of different treatments within the same patient over time, and enabling the study of tumours with varying histological characteristics within the same study. This could be one approach to generate comparative efficacy data for a drug developed in single-arm trials [6–8]. Using the GMI to make an early assessment of treatment efficacy is appealing because it could achieve the dual goals of having a controlled evaluation based on TTP and a single treatment group design [6, 9].

GMI has recently gained in popularity in the scientific literature but few data regarding its use are available. To the best of our knowledge, there is no standardized definition for this outcome. There is variability across publications in terms of both definitions and cut-offs used to assess efficacy, leading to a lack of comparability and reproducibility between studies. Moreover, the GMI has been recently criticized, especially its arbitrary 1.3-cut-off used to define treatment efficacy [10]. To date, there is no review synthesizing the different uses of GMI in the literature nor trying to develop guidelines or recommendations for good practices about its use. Our aim was to map the literature on the GMI in order (i) to describe its use in the clinical studies, and (ii) to synthesize the methodological advances.

## Material and Methods

We conducted a scoping review to investigate the evidence on the GMI.

### Selection of Manuscripts

We performed a review through a computerized search of databases to identify literature based on GMI. We investigated the following databases: PubMed, Scopus, Web of Science and BASE (Bielefeld Academic Search Engine). The search algorithms included the following key words: growth modulation index, time to progression ratio and progression-free survival (PFS) ratio. Full search queries are provided (Supplemental Table S1). Additional cross-referencing from reference lists of relevant articles was performed. We included documents published up to the 31st December 2023, with a full text available in English. We excluded preprints. Based on the full text, we retrieved those documents that calculated and/or commented on the GMI. We relied on the Rayyan application in order to combine the search results from the databases and remove duplicates [11]. When a congress abstract and/or a PhD thesis was available in addition to the associated manuscript, only the manuscript was retained. If several manuscripts reported on the same study, only the most recent publication was retained. Finally, if both the protocol and the results had been published for the same study, we only retained the publication pertaining to the results.

### Data Extraction

All authors participated in the development of the standardized data extraction grid (Supplemental Table S2). This grid was independently completed for each publication by two reviewers (KT + CD, DD or CB). Disagreements were resolved by consensus between the two concerned reviewers. We collected information about study design and participants (observational/interventional study, retrospective/prospective study, unicentric/multicentric study, eligibility criteria), randomization and masking if applicable, studied procedure(s) (type of treatments), outcome (follow-up frequency and duration, definition of PFS/TTP) and statistical analysis (cut-off for GMI, missing data, type of statistical analysis, graphical representation, sample size determination, number of included subjects). To properly define the GMI, one must clearly state the “Time 0” for PFS/TTP for both lines, as well as events considered in the definitions of PFS/TTP. These elements were therefore extracted from the documents. For those manuscripts reporting specifically on methodological or statistical issues regarding the GMI, we collected information regarding the aim of the manuscript (GMI modelling, statistical testing, sample size estimation) and statistical methods (parametric, non-parametric). We used the REDCap software to collect data from the assessed documents [12, 13].

### Statistical Analysis

We reported descriptive statistics. We relied on the R statistical software for both statistical analysis and data visualization (version 4.4.0) [14].

## Results

Results are reported following the Preferred Reporting Items for Systematic reviews and Meta-Analyses extension for Scoping Reviews (PRISMA-ScR) guidelines (Supplemental Table S3) [15].

### Eligible Documents

**Fig. 1:**
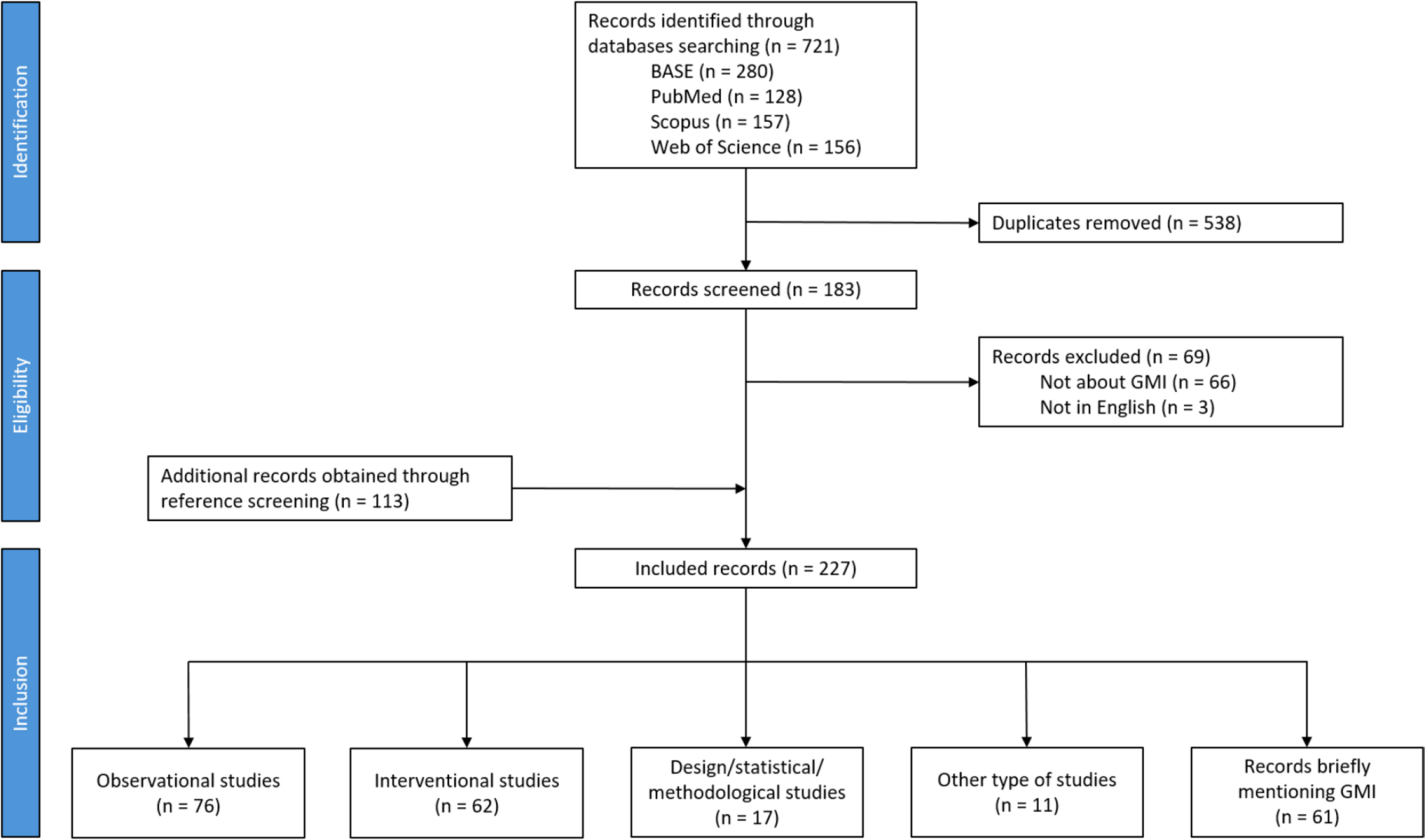
Flow chart of studies included in the scoping review

Our search equations identified 721 documents. Once duplicates were removed, we ended up with 183 unique documents (Fig. 1). We excluded 69 documents: 66 documents did not report on the GMI and for 3 documents, the full text was not available in English. We further identified 113 documents through cross-referencing. In total, we included 227 documents in our scoping review. We retrieved 211 (93.0%) articles, 14 (6.2%) conference/congress abstracts, 1 (0.4%) book chapter, and 1 (0.4%) PhD thesis.

Sixty-one (26.9%) of the 227 included documents were considered as records briefly mentioning GMI [16–76]. These documents briefly cited GMI results from other studies, without reporting their own GMI data/statistics, nor discussing GMI-based methods.

These documents will thus not be further discussed (Supplemental Table S4). We focus below on the remaining 166 documents.

### General Characteristics of the Included Documents

**Fig. 2:**
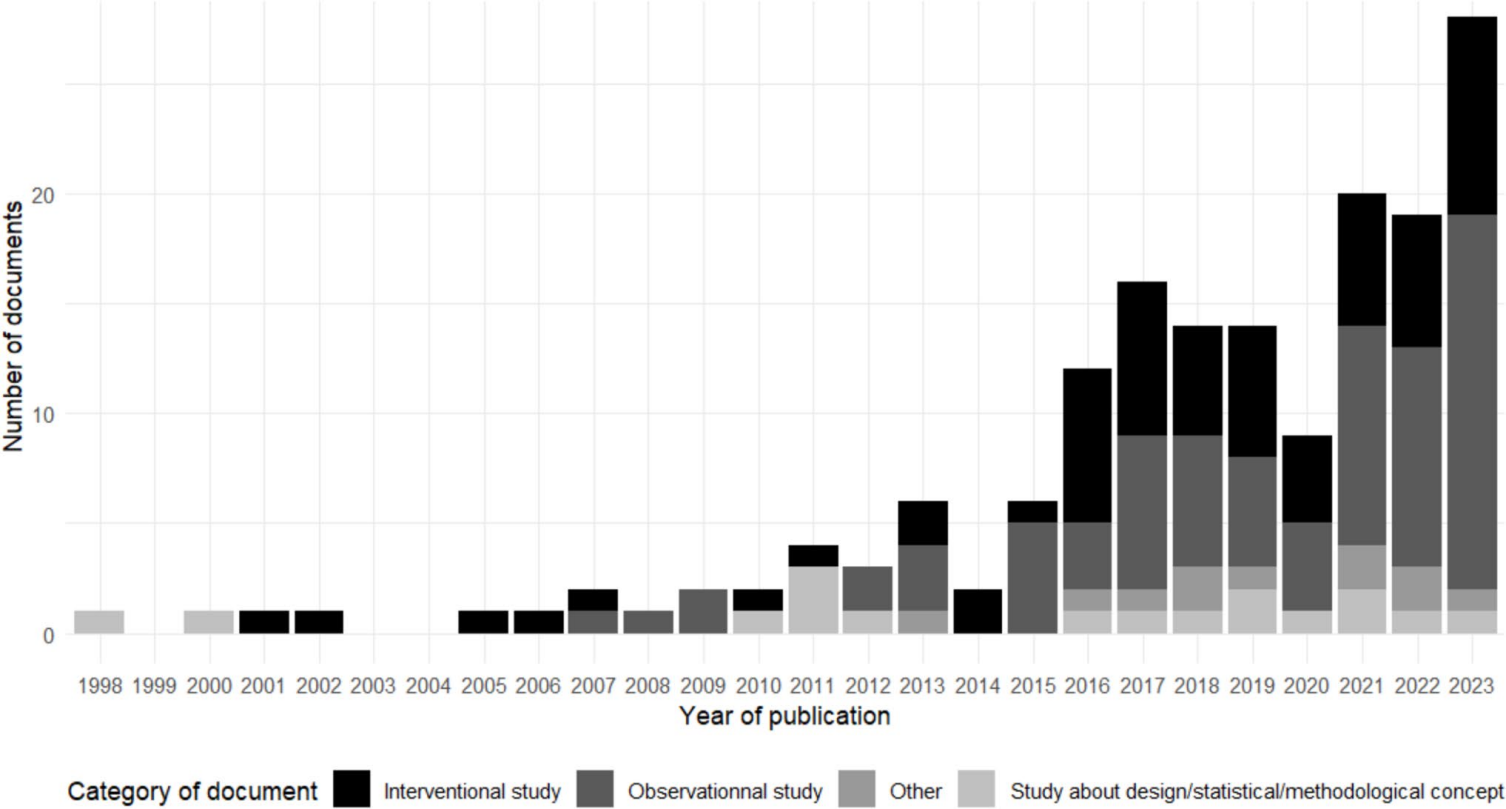
Number of original articles about GMI published per year

Since the original 1998 manuscript introducing the GMI [1], the number of yearly publications reporting on GMI data and/or methods increased over time (Fig. 2).

Among the 166 included documents, authors referred to the GMI using various terminologies: “PFS ratio” (*n* = 99, 59.6%), “GMI” (*n* = 66, 39.8%), “TTP ratio” (*n* = 17, 10.2%), “Von Hoff ratio/analysis/criterion/model” (*n* = 6, 3.6%) and other terminologies such as “Time to failure ratio” and “TTP index” (*n* = 5, 3.0%). In some cases, several terms were used in the same document (*n* = 26, 15.7%).

Documents focused only on oncology: either solid tumours (*n* = 131, 94.9%), blood cancers (*n* = 5, 3.6%) or both (*n* = 2, 1.4%). Most frequent countries of the first affiliation of the first author were USA (*n* = 40, 24.1%) and France (*n* = 37, 22.3%).

Most of the 166 documents reported results of observational studies (*n* = 76, 45.8%) [5, 7, 9, 77–149] or interventional studies (*n* = 62, 37.3%) (Fig. 1, Supplemental Tables S5 and S6) [2, 6, 8, 150–208]. In addition, 17 (10.2%) documents reported on methodological and/or statistical developments pertaining to the GMI [1, 3, 4, 10, 209–221]. The rest of the documents (*n* = 11, 6.6%) were other types of documents such as case reports or case series [222–232].

### Observational and Interventional Studies

Out of the 76 observational studies, most were retrospective studies (85.5%) (Table 1). The 62 interventional studies were mainly phase II trials (*n* = 49, 79.0%).

**Table 1:** Summary of characteristics of documents reporting observational or interventional studies

	Interventional studies (*n* = 62)	Observational studies (*n* = 76)	Total (*n* = 138)
Design			
Phase I	6 (9.7%)	-	6 (4.3%)
Phase II	49 (79.0%)	-	49 (35.5%)
Phase III	5 (8.1%)	-	5 (3.6%)
Phase IV	1 (1.6%)	-	1 (0.7%)
Other	1 (1.6%)	-	1 (0.7%)
Retrospective study	-	65 (85.5%)	65 (47.1%)
Prospective study	-	11 (14.5%)	11 (8.0%)
Outcome			
Primary outcome	23 (37.1%)	24 (31.6%)	47 (34.1%)
Secondary/Exploratory outcome	39 (62.9%)	52 (68.4%)	91 (65.9%)
Start of previous line			
Initiation of treatment line	26 (41.9%)	42 (55.3%)	68 (49.3%)
Randomization	3 (4.8%)	0 (0%)	3 (2.2%)
Enrollment	1 (1.6%)	0 (0%)	1 (0.7%)
Not defined	32 (51.6%)	34 (44.7%)	66 (47.8%)
Events considered for progression during previous line			
Progression (not otherwise specified)	29 (46.8%)	30 (39.5%)	59 (42.8%)
Radiological progression	17 (27.4%)	21 (27.6%)	38 (27.5%)
Clinical progression	3 (4.8%)	10 (13.2%)	13 (9.4%)
End of treatment	3 (4.8%)	2 (2.6%)	5 (3.6%)
Initiation of next line	2 (3.2%)	2 (2.6%)	3 (2.2%)
Toxicity	0 (0%)	1 (1.3%)	1 (0.7%)
Treatment failure	0 (0%)	1 (1.3%)	1 (0.7%)
Last visit	0 (0%)	1 (1.3%)	1 (0.7%)
Not defined	14 (22.6%)	23 (30.3%)	37 (26.8%)
Start of next line			
Initiation of treatment line	29 (46.8%)	46 (60.5%)	75 (54.3%)
Date of cross-over	4 (6.5%)	0 (0%)	4 (2.9%)
Study entry	3 (4.8%)	0 (0%)	3 (2.2%)
Biomarker analysis	0 (0%)	1 (1.3%)	1 (0.7%)
Day after first-line progression	1 (1.6%)	0 (0%)	1 (0.7%)
Molecular profiling request	0 (0%)	1 (1.3%)	1 (0.7%)
End of previous line	0 (0%)	1 (1.3%)	1 (0.7%)
Relapse from previous treatment	1 (1.6%)	0 (0%)	1 (0.7%)
Not defined	24 (38.7%)	27 (35.5%)	51 (37.0%)
Events considered for progression during next line			
Progression (not otherwise specified)	29 (46.8%)	33 (43.4%)	62 (44.9%)
Radiological progression	20 (32.3%)	22 (28.9%)	42 (30.4%)
Clinical progression	4 (6.5%)	10 (13.2%)	14 (10.1%)
Death	27 (43.5%)	29 (38.2%)	56 (40.6%)
Last visit	2 (3.2%)	5 (6.6%)	7 (5.1%)
End of treatment	2 (3.2%)	2 (2.6%)	4 (2.9%)
Initiation of next line	3 (4.8%)	1 (1.3%)	4 (2.9%)
Toxicity	0 (0%)	2 (2.6%)	2 (1.4%)
Removal of the study	1 (1.6%)	0 (0%)	1 (0.7%)
Not defined	13 (21.0%)	19 (25.0%)	32 (23.2%)
GMI cut-off			
0.7	3 (4.8%)	0 (0%)	3 (2.2%)
0.8	0 (0%)	1 (1.3%)	1 (0.7%)
1	10 (16.1%)	11 (14.5%)	21 (15.2%)
1.1	1 (1.6%)	0 (0%)	1 (0.7%)
1.2	2 (3.2%)	0 (0%)	2 (1.4%)
1.25	1 (1.6%)	0 (0%)	1 (0.7%)
1.3	38 (61.3%)	48 (63.2%)	86 (62.3%)
1.33	14 (22.6%)	18 (23.7%)	32 (23.2%)
1.42	1 (1.6%)	0 (0%)	1 (0.7%)
1.5	4 (6.5%)	1 (1.3%)	5 (3.6%)
1.8	1 (1.6%)	0 (0%)	1 (0.7%)
2	1 (1.6%)	2 (2.6%)	3 (2.2%)
2.5	1 (1.6%)	0 (0%)	1 (0.7%)
5	0 (0%)	1 (1.3%)	1 (0.7%)
No cut-off used	2 (3.2%)	6 (7.9%)	8 (5.8%)

Most of the 138 observational and interventional studies involved multiple solid tumours (*n* = 52, 37.7%). When a single indication was assessed, this was most often soft-tissue sarcoma (*n* = 15, 10.9%) or breast cancer (*n* = 11, 8.0%). A total of 115 studies (83.3%) reported on advanced/metastatic setting. For 16 studies, the setting was not clearly stated (11.6%).

Half of the 138 observational and interventional studies evaluated sequence-guided strategies (*n* = 69, 50.0%). Twenty-six studies (18.8%) and 39 studies (28.3%) evaluated fixed combinations of treatment and single treatment, respectively. The previous line of treatment was defined in 112 studies (81.2%) and not defined or not clear in 26 studies (18.8%). A wash-out period between the treatment lines was described in few articles (*n* = 3, 2.2%), all of them were interventional studies.

GMI was most often reported as a secondary or exploratory outcome in 92 documents (66.7%), and as a primary outcome in 46 documents (33.3%). Time 0 for PFS/TTP for both lines was most often the initiation of the treatment line (*n* = 68, 49.3% for the previous line and *n* = 75, 54.3% for the second line) (Table 1). Time 0 for the previous and next lines was however not described in 66 documents (47.8%) and 51 documents (37.0%), respectively. End of both lines was most often progression without additional detail on how this progression was evaluated (*n* = 59, 42.8% for the previous line and *n* = 62 studies, 44.9% for the second line). Thirty-seven documents (26.8%) did not describe events considered for the end of the previous line, and 32 documents (23.2%) for the next line.

There was a variety of GMI cut-offs used to assess treatment efficacy, ranging from 0.7 to 5, with sometimes several cut-offs investigated in the same document (Table 1). The 1.3-cut-off was used most frequently (*n* = 86, 62.3%), followed by 1.33, as originally proposed by Von Hoff (*n* = 32 studies, 23.2%). In 8 studies (5.8%), the cut-off was not clearly defined. In the 46 studies with GMI as the primary outcome, the sample size determination was based on the estimation of a proportion of patients with a GMI superior to a specific cut-off (*n* = 17, 37.0%). We identified no other approach used for the sample size determination of GMI-based studies. The GMI was usually reported in terms of a proportion of patients presenting a GMI above a specified cut-off (*n* = 108, 78.3%) (Table 1). The median GMI could also be reported (*n* = 56 studies, 40.6%), as well as the range (*n* = 42, 30.4%) or a confidence interval (*n* = 23, 16.7%). Description of time until both previous and next line progression is reported in 51 studies (37.0%), only for previous line progression in two studies (1.4%), only for next line in 35 studies (25.4%), and neither in 50 studies (36.2%). The GMI was presented graphically in 64 studies (46.4%). The most frequent graphical displays included bar plots/swimmer plots (*n* = 51, 79.7%) and Kaplan–Meier plots (*n* = 9, 14.1%).

### Methodological and Statistical Developments Pertaining to the GMI

Among the 17 documents retrieved (Supplemental Table S7), 11 (64.7%) documents discussed general concepts related to the GMI, 5 (29.4%) reported on statistical methods for GMI modelling and 1 (5.9%) reported on GMI-based sample size determination. Among the five studies reporting statistical methods for GMI modelling, three of them (60.0%) proposed parametric and non-parametric approaches [3, 211, 213] and two (40.0%) proposed a parametric approach [4, 210]. Non-parametric approaches were multiple and included GMI modelling based on count of GMI outcomes equal to or greater than a prespecified cut-off, methods based on the use of ranks of the paired TTP/PFS times (midrank), on the Kaplan–Meier (KM) estimator and on a kernel conditional KM estimator. Among the parametric approaches, we identified various methods for GMI modelling including survival frailty models according to a Weibull distribution, or modelling according to a bivariate lognormal distribution or to a log-linear distribution. One publication reported on methods for GMI-based sample size [10]. The sample size formula was derived from a log-linear model for paired survival difference and illustrated under a bivariate exponential model, a Weibull frailty model and a generalized treatment effect size.

## Discussion

### Summary of Results

To the best of our knowledge, this is the first review investigating the use of the GMI in the literature. We highlighted an increasing trend over years in the use of the GMI, whether it relates to the reporting of clinical studies or to methodological publications. The terminology used to refer to the GMI was highly heterogeneous, as additional terms such as PFS or TTP ratio were often used. In addition, TTP and PFS may be (incorrectly) used synonymously. While TTP is defined as the time to objective tumour progression and excludes death, PFS corresponds to the time to objective tumour progression or death (any) [233]. So, in theory, when one is interested in investigating a treatment for a subsequent line, the PFS of the next treatment is compared to the TTP under the preceding line. Very few publications relied on this appropriate definition. Terms such as PFS ratio or TTP ratio should thus be avoided and replaced by GMI. Next, irrespective of the terms employed to refer to GMI, information is often lacking with regards to the definition of GMI, such as the initial time used under previous and/or next lines, and/or event considered in the TTP/PFS outcomes, coherent with past publication reporting on the poor quality of the reporting of survival outcomes [234]. Finally, information regarding the sample size of the population used for the computation of the GMI was not systematically reported, and the presence/absence of a wash-out period was almost never mentioned. There is a need for standardization when reporting on the GMI, in terms of both terminology and definition.

### GMI Properties

Using the GMI as an efficacy outcome can be particularly attractive. As it relies on an intra-patient comparison, both known and unknown individual confounding variables (e.g. age, sex, presence of genomic mutations, etc.) are controlled for [6, 107]. In simulation studies, this has been shown to lead hypothetically to better statistical power compared to parallel-arm groups (and thus smaller sample size), which is especially true as the correlation between the time until progression of the two treatment lines increases [4, 217]. The European Medicines Agency (EMA) considers that in previously treated patients, a within patient comparison of TTP/PFS might provide evidence of activity [235]. However, GMI can only be used for patients who have failed a first-line treatment. It cannot be used to assess the efficacy of a first-line treatment. In such situations, a trial design with a comparator arm is required for treatment evaluation. It should be noted, also, that GMI requires that tumour growth kinetics is linear over time, even if a linear evolution does not match tumour models [6, 214]. This linearity assumption is difficult to assess. However, assuming linear tumour growth may be more acceptable in the advanced setting as time to progression may be particularly short as compared to the adjuvant setting. For exploratory purposes however, this constitutes no major concern [235]. Both the schedule and method for outcome assessment should be the same between the two treatment lines in order to ensure that assessment of progression is comparable between lines [3, 214]. This assumption does not hold usually for retrospective studies. Moreover, in the context of prospective trials, patients are usually included following treatment failure. While schedule for outcome assessment under experimental treatment is usually precisely defined in the study protocol, timing of the assessment under the preceding line, i.e. before trial entry, can be highly variable. GMI seems to be associated with classical activity outcomes (such as best objective response and response at 3 or 6 months) and correlated with overall survival (OS) [2, 88, 90, 159, 226]. We can consider that GMI is an appropriate outcome for evaluating tumour burden process but only as a proxy of clinical benefit. When possible, OS is the preferred outcome to adequately assess clinical benefit of a new treatment [233]. GMI does not always align with physician perception, particularly in cases of rapid progression where small absolute gains in PFS/TTP might be deemed insufficient despite a GMI above the threshold. Conversely, in cases of slow progression, a GMI below the threshold may not reflect clinical satisfaction with treatment efficacy. To address this, Mock et al. proposed a modified GMI, adjusting for extreme PFS/TTP values, which has shown varying levels of agreement with the original GMI [122, 158, 220]. Further research is needed to determine if this modified approach should replace Von Hoff's original GMI.

### Strengths of the Review

We consulted four databases to retrieve documents, including one for grey literature. Each document was read by two reviewers, who relied on a standardized reading grid. As our primary aim was to investigate how the GMI was reported in the literature (rather to report a summary measure of some treatment effect), we did not assess the quality of included documents, which is not mandatory for a scoping review [15].

### Limitations of the Review

Our search algorithm included several terms used to refer to the GMI, and we also relied on cross referencing. As the GMI is often reported as a secondary efficacy measure, it may not be available in the abstract, and can be relegated to either full text, tables or supplementary material. It thus remains possible that our search procedure missed some publications.

### Key Elements for an Appropriate GMI Reporting

As the number of studies reporting GMI results is increasing, it appears necessary to develop a framework for improving the quality and transparency of publication reporting GMI. To this extent and based on a philosophy similar to the CONSORT statement, we identified key elements that we think should be provided for the readers to properly assess published GMI results (Table 2). For ease of exposition, we provided some illustrative examples, extracted from publications retrieved during our review.

**Table 2:** Proposed recommendations when reporting GMI

	Recommendation	Example
Methods: outcome	Prefer the term “GMI” as originally defined, and define it precisely	« GMI was defined as the ratio of PFS on larotrectinib to TTP on the most recent prior line of therapy on which the patient had just progressed (Von Hoff 1998).» [9]
	Properly define the numerator and the denominator, i.e. for each: - Define time 0, - Define events considered	« PFS was defined as the time from larotrectinib initiation to radiological progression (Response Evaluation Criteria in Solid Tumors (RECIST) v1.1 per independent review committee (IRC)), clinical progression, or death from any cause. […] TTP was defined as the time from the start of the last prior treatment to radiological progression (RECIST v1.1), treatment failure, or clinical progression» [9]
Methods: design	Define the treatment(s) for prior and next lines	« GMI was defined as the ratio of PFS on larotrectinib to TTP on the most recent prior line of therapy on which the patient had just progressed» [9]
	Report the schedule and the modality of outcome assessment for each line If not done or impossible (ex: retrospective data), detail why	« In TTP1, tumor assessments were carried out at baseline, 4 weeks after baseline and every 6 weeks subsequently. In the case of obvious clinical progression during TTP1, a computed tomography (CT) scan was carried out immediately. […] In TTP2, tumor assessments were carried out every 8 weeks until progressive disease, according to RECIST, was observed. The only exception was patients who were already progressive in TTP1 at 4 weeks, they had their first on-treatment scan at 4 weeks.» [159]
	Indicate whether there was a wash-out period	« The second line of treatment could only start if the patient had clinically and biologically recovered from first-line treatment toxicities, and after a wash out of at least 3 weeks since the last dose of chemotherapy» [166]
Results: analysis	Properly define the subgroup of patients used for the computation of the GMI	« The Von Hoff ratio (PFS2/PFS1) was also determined for the patients receiving molecularly guided therapy in the second or later-line setting. Thirty-eight percent (3 of 8) had a molecularly guided treatment with a duration that was ≥ 1.3 times the prior line of therapy» [97]
	Report the median PFS/TTP of each treatment line and the associated 95% confidence interval and provide sample size	« For all patients (*n* = 72), median TTP on the previous line of treatment was 3.0 months (95% CI, 2.6–4.4) and median PFS on larotrectinib was not estimable (NE) (95% CI, NE; hazard ratio (HR), 0.220 (95% CI, 0.146–0.332)» [9]
	Report a summary statistic of GMI + a measure of statistical dispersion (median + IQR or min–max range)	« The median GMI was 0.75 (range, 0.01–27.5).» [88]
	Report the number and the percentage of subjects above the GMI cut-off(s) and the number of subjects for which the GMI was calculated	« Thirty of 56 patients (54%; 95% confidence interval, 40%–67%) from the primary analysis set reached a PFS ratio (PFS on scFPM-guided therapy compared with PFS on prior therapy) of ≥ 1.3 with a median PFS ratio of 3.4 [interquartile range (IQR), 2.2–5.7].» [176]

*GMI* Growth modulation index, *HR* Hazard ratio, *IQR* Interquartile range, *NE* Not estimable, *PFS* progression free−survival, *RECIST* Response evaluation criteria in solid tumors, *TTP* time to progression

### Perspectives

At the time of this review, we found no work validating an optimal cut-off for GMI. Originally, Von Hoff set an arbitrary cut-off at 1.33, which would be used in many studies thereafter [1]. However, some authors considered that a cut-off at 1, expressing a similar TTP/PFS between the two lines, is sufficient to show the superiority of the next line [3]. Cut-offs used should be validated and justified. It is not clear however if this cut-off should be universal or specific to some situations (e.g. dependent on the treatment mechanism of action or tumour histology for example) and if sensitivity analyses with different cut-offs should be done [145]. These developments are important to avoid biased interpretations of clinical results and potentially inappropriate therapeutic decisions.

Among the 47 studies using on GMI as a primary outcome, 18 based the sample size on the estimation of a proportion of patients with a GMI greater than some specific threshold, leading to loss of information, with most studies not justifying their assumptions [5]. This binary approach ignores that the GMI is made up of right-censored data [3, 211, 213]. We identified only one statistical work about sample size determination where the GMI was actually considered as a ratio of survival outcomes [10]. Statistical methods used for handling the GMI were highly variable, and both parametric and non-parametric approaches have been investigated. In practice however, we did not identify any prospective trial that relied on a sample size estimation process based on GMI modelling. The diversity of the statistical methods employed for the modelling of the GMI, but also the scarcity of such methodological GMI-based works is partly explained by the statistical nature of this criterion. The GMI is a ratio of time-to-event outcomes which thus is complex to model. It is overly difficult to postulate a statistical distribution for this outcome, and thus to derive statistical tests and sample size methods based on this parameter. Further statistical developments are needed to obtain and spread sample size formulas for studies based on GMI and to properly estimate this parameter.

## Conclusion

To the best of our knowledge, this is the first review investigating the use of the GMI in the literature. We highlighted important heterogeneity in the reporting of GMI, and thus proposed a set of key items to provide when reporting GMI data. Although the use of this efficacy outcome has been increasing over the recent years, methodological and statistical development are needed to properly model GMI and assess relevant GMI thresholds to define efficacy. This could be an opportunity to investigate GMI-based trial designs, particularly for phase II single-arm cancer trials.

## Key References

- Van De Kruis N, Van Der Ploeg P, Wilting JHC, Caroline Vos M, Thijs AMJ, De Hullu J, et al. The progression-free survival ratio as outcome measure in recurrent ovarian carcinoma patients: Current and future perspectives. Gynecol Oncol Rep. 2022;42:101035.
  - ○ This study examines the factors associated with the GMI value in recurrent ovarian carcinoma patients. It highlights the challenge of defining a clinically relevant GMI threshold to consider efficacy. As such, the manuscript underscores the need for further evaluation, based on clinical data, to establish a valid GMI threshold for future studies.
- Du Rusquec P, Guimbaud R, Le Malicot K, Gornet J-M, Nguyen S, Lecomte T, et al. Evaluation of the relevance of the growth modulation index (GMI) from the FFCD 0307 randomized phase III trial comparing the sequence of two chemotherapeutic regimens. ESMO Open. 2023;8:101616.
  - ○ This study re-analyzes a clinical trial by assessing efficacy using the GMI. It compares the GMI-based conclusion to those relying on overall survival or progression-free survival. It questions the relevance of the GMI as an outcome in oncology trials.
- Samaille T, Moreau Bachelard C, Coquan E, du Rusquec P, Paoletti X, Le Tourneau C. Impact of the timing of tumor assessments on median progression-free survival in clinical trials in advanced cancer patients. ESMO Open. 2022;7:100366.
  - ○ This study examines the impact of tumor assessment timing on the determination of progression-free survival and the potential for evaluation-time bias in clinical research. When assessing treatment efficacy based on the GMI, the study emphasizes the importance of maintaining the same schedule of assessments, for a given patient, between successive treatment lines.
- Chen L, Burkard M, Wu J, Kolesar JM, Wang C. Estimating the distribution of ratio of paired event times in phase II oncology trials. Stat Med. 2023;42:388–406.
  - ○ This simulation study proposes and evaluates the latest parametric and non-parametric approaches for GMI modeling, accounting for its dependent censoring.
- European Medicines Agency. Guideline on the Clinical Evaluation of Anticancer Medicinal Products. Available at https://www.ema.europa.eu/en/documents/scientific-guideline/guideline-clinical-evaluation-anticancer-medicinal-products-revision-6_en.pdf. Accessed July 5, 2024.
  - ○ To our knowledge, this is the first and only regulatory agency guideline to mention the GMI as a potential outcome for evaluating treatment activity in phase II exploratory studies.

## Supplementary Information

Below is the link to the electronic supplementary material.

Supplementary file1 (DOCX 14 KB)

Supplementary file2 (DOCX 28 KB)

Supplementary file3 (DOCX 54.2 KB)

Supplementary file4 (DOCX 59 KB)

Supplementary file5 (DOCX 79 KB)

Supplementary file6 (DOCX 82 KB)

Supplementary file7 (DOCX 25 KB)

Supplementary file8 (DOCX 23 KB)

## Data Availability

Any data (protocol, data set or statistical code) can be supplied on reasonable request by contacting the corresponding authors.
