# Supplementary material for: The Growth Modulation Index (GMI) as an Efficacy Outcome in Cancer Clinical Trials: A Scoping Review with Suggested Reporting Guidelines
Source: Curr Oncol Rep. 2025 Mar 29;27(5):516–32. doi: 10.1007/s11912-025-01667-1 (PMC12081581; doi:10.1007/s11912-025-01667-1)
Supplement: Supplementary file 1 — Supplementary file1 (DOCX 14 KB) [file 11912_2025_1667_MOESM1_ESM.docx]

**Table S1** Search query for each base used in the scoping review and number of associated results

| Base | Search Query | Results |
| --- | --- | --- |
| PubMed | ((((((("growth modulation index"[Title/Abstract]) OR ("time to progression ratio"[Title/Abstract])) OR ("progression free survival ratio"[Title/Abstract])) OR ("PFS2/PFS1"[Title/Abstract])) OR ("PFS ratio"[Title/Abstract])) OR ("ratio of time to progression"[Title/Abstract])) OR ("ratio of progression-free survival"[Title/Abstract])) OR ("TTP ratio"[Title/Abstract]) | 128 |
| Scopus | (TITLE-ABS-KEY ("growth modulation index") OR TITLE-ABS-KEY ("time to progression ratio") OR TITLE-ABS-KEY ("progression free survival ratio") OR TITLE-ABS-KEY ( "PFS2/PFS1") OR TITLE-ABS-KEY ("PFS ratio") OR TITLE-ABS-KEY ("ratio of progression-free survival") OR TITLE-ABS-KEY ("ratio of time to progression") OR TITLE-ABS-KEY ("TTP ratio")) | 157 |
| Web of Science | (((((((ALL=("growth modulation index")) OR ALL=("progression free survival ratio")) OR ALL=("time to progression ratio")) OR ALL=("PFS2/PFS1")) OR ALL=("PFS ratio")) OR ALL=("ratio of time to progression")) OR ALL=("ratio of progression-free survival")) OR ALL=("TTP ratio") | 156 |
| Bielefeld Academic Search Engine | ("growth modulation index" "progression free survival ratio" "time to progression ratio" "PFS2/PFS1" "PFS ratio" "ratio of progression-free survival" "ratio of time to progression" "TTP ratio") | 280 |
