# Supplementary material for: The Growth Modulation Index (GMI) as an Efficacy Outcome in Cancer Clinical Trials: A Scoping Review with Suggested Reporting Guidelines
Source: Curr Oncol Rep. 2025 Mar 29;27(5):516–32. doi: 10.1007/s11912-025-01667-1 (PMC12081581; doi:10.1007/s11912-025-01667-1)
Supplement: Supplementary file 2 — Supplementary file2 (DOCX 28 KB) [file 11912_2025_1667_MOESM2_ESM.docx]

**Table S2** Standardized data extraction grid

| ***Manuscript information*** |
| --- |

| Study ID | *Incremental REDCap n°* |
| --- | --- |
| Last name first author | *Text* |
| Full title | *Text* |
| Year of publication | *4-digit integer* |
| Type of scientific literature | *One answer*  Peer-review journal  Conference/Congress abstract  PhD thesis  Other, specify: *text* |
| If peer-review journal, name of the journal: | *One answer*  American Health & Drug Benefits  Annals of Oncology  Annals of Translational Medicine  Anti-cancer Drugs  BMC Cancer  Breast  Breast Cancer Research and Treatment  British Journal of Cancer  Bulletin du Cancer  Cancer Diagnosis & Prognosis  Cancer Discovery  Cancer Research  Cancer Research and Treatment  Cancers  Clinical & Transnational Oncology  Clinical Cancer Research  Computational and mathematical methods in medicine  Contemporary clinical trials  Controlled Clinical Trials  Current Oncology  Current Opinion in Oncology  Current Pharmaceutical Design  Diagnostics  Drug Design Development and Therapy  EBioMedicine  ESMO Open  European Journal of Cancer  Future Oncology  Gastroenterologie Clinique et Biologique  Genome Medicine  Gynecologic Oncology Reports  International Journal of Radiation Oncology Biology Physics  Journal of Cancer Research and Clinical Oncology  Journal of Clinical Oncology  Journal of Haematology & Oncology  Journal of Neuro-Oncology  Molecular Cancer Therapeutics  Molecular Oncology  Nature Medicine  Neuro-oncology Advances  NPJ Breast Cancer  Oncotarget  Pancreas  Pharmaceutical Statistics  Scientific Reports  Statistics in Medicine  Targeted Oncology  The Lancet Haematology  Therapeutic Advances in Medical Oncology  Other, specify: *Text* |
|  | |
| ***First reviewer*** | |
| Reviewer | *One answer*  Kilian TRIN |
| **Eligibility criteria** | |
| GMI or it(s) equivalent(s) calculated and/or commented on | *One answer*  Yes  No |
| GMI or it(s) equivalent(s) calculated and/or commented on | *One answer*  Yes  No |
| Full text available | *One answer*  Yes  No |
| Full text available in English? | *One answer*  Yes  No |
| If “Full text available in English = No”, language | French  Korean  Turkish  Other, specify: *Text* |
| **If one « no », stop the completion of the grid* | |
| **General informations** | |
| First affiliation of first author | *One answer*  Academic  Industrial  Academic and industrial  Not clear, specify: *Text* |
| Country of the first author (*use the ISO 3166-1 alpha-3 country classification; if several countries, select the country of the first mentioned affiliation*) | *Text (3 letters)* |
| Medical field(s) covered | *Several answers possible*  Oncology  Infectious diseases  Cardiovascular diseases  Other field, specify: *text*  Not specified |
| Terminology used | *Several answers possible*  Growth modulation index / GMI  Time to progression ratio / TTP ratio  Progression free survival ratio / PFS ratio  Other, specify: *text* |
| Topic covered by the manuscript | *One answer*  Publication of the results of an observational study  Publication of the results of an interventional study  Publication on design/statistical/methodological concept  Other, specify: *text* |
| **If “Publication of the results of an observational study” OR “Publication of the results of an interventional study”:** | |
| **Study design and participants** | |
| Multicenter study? | *One answer*  Yes, 2 countries or more  Yes, 1 country  No (one center)  No information provided |
| Study design | *One answer*  Observational prospective study  Observational retrospective study  Observational study, other/specify:  Interventional phase I  Interventional phase II / single-arm trial  Interventional phase II / basket  Interventional phase II / umbrella  Interventional phase II / other multi-arm biomarker-guided trial  Interventional phase III  Interventional phase IV  Other, specify: *text* |
| Randomization | *One answer*  Yes  No |
| Indication | *Several answers possible*  Oncology, multiple solid tumors  Oncology, biliary tract  Oncology, breast  Oncology, cervix  Oncology, colorectal cancer  Oncology, gastric/oesogastric junction  Oncology, head and neck  Oncology, kidney  Oncology, lung cancer  Oncology, melanoma  Oncology, ovary  Oncology, pancreas  Oncology, prostate  Oncology, soft-tissue sarcoma  Oncology, urothelial  Oncology, uterus  Oncology, other, specify: *text*  Not oncology, specify: *text* |
| **Intervention** | |
| Setting for oncology studies | *One answer*  Neoadjuvant  Adjuvant  Advanced/Metastatic  Not clear/Not defined  Other, specify: *text* |
| Treatment(s) first-line | *One answer*  Combination of treatments  One treatment  Not clear/Not defined |
| If “Treatment(s) first-line = Combination of treatments”, treatments: | *Several answers possible*  Targeted therapy  Immunotherapy  Cytotoxic therapy  Hormonal therapy  Radiotherapy  Other, specify: *text* |
| If “Treatment(s) first-line = One treatment”, treatment: | *One answer*  Targeted therapy  Immunotherapy  Cytotoxic therapy  Hormonal therapy  Radiotherapy  Other, specify: *text* |
| Treatment(s) next-line | *One answer*  Combination of treatments  One treatment  NGS-guided treatment  Not clear/Not defined |
| If “Treatment(s) next-line = Combination of treatments”, treatments: | *Several answers possible*  Targeted therapy  Immunotherapy  Cytotoxic therapy  Hormonal therapy  Radiotherapy  Other, specify: *text* |
| If “Treatment(s) next-line = One treatment”, treatment: | *One answer*  Targeted therapy  Immunotherapy  Cytotoxic therapy  Hormonal therapy  Radiotherapy  Other, specify: *text* |
| Wash-out period | *One answer*  Yes, duration in weeks: *2-digit integer*  No  Not clear/Not defined |
| **Outcome** | |
| GMI/ratio | *One answer*  Primary endpoint  Secondary/exploratory endpoint |
| Numerator of the GMI/ratio - Time 0 | *One answer*  Initiation of treatment line  Other, specify:  Not defined |
| Numerator of the GMI/ratio - Events considered | *Several answers possible*  Clinical progression  Radiological progression  Progression (not otherwise specified)  Toxicity  End of treatment  Death  Other, specify: *text*  Not defined |
| Denominator of the GMI/ratio - Time 0 | *One answer*  Randomization  Initiation of treatment line  Other, specify: *text*  Not defined |
| Denominator of the GMI/ratio - Events considered | *Several answers possible*  Clinical progression  Radiological progression  Progression (not otherwise specified)  Toxicity  End of treatment  Other, specify: *text*  Not defined |
| **Statistical analysis** | |
| GMI cut-off used | *One answer*  Yes, specify: *(0 – 99, with two decimals)*  No |
| If GMI = primary endpoint: Sample size determination based on GMI | *One answer*  Yes, based on a proportion of GMI > defined cut-off  Yes, not based on a proportion, specify: *text*  No |
| **Results** | |
| Number of subjects included | *Integer (0-9999)* |
| Number of subjects used for GMI computation was reported | *One answer*  Yes, specify: *Integer (0-9999)*  No |
| Median PFS/TTP 1^st^ line | Yes, specify (months): *(0 – 99, with one decimal)*  No |
| Median PFS/TTP next line | Yes, specify (months): *(0 – 99, with one decimal)*  No |
| Descriptive statistics for GMI | Yes  No |
| If “Descriptive statistics for GMI = Yes”, Specify | Median  Interquartile range  Range (min-max)  Mean  Standard deviation  Proportion  Confidence interval  Other, specify: *text* |
| Graphical representation for GMI | Yes, specify: *text*  No |
| **If “Publication on design/statistical/methodological concept”:** | |
| Objective of the manuscript | *Several answers possible*  Discussion/concept  Statistical methods for GMI modeling  Parametrical  Non parametrical  Statistical methods for GMI hypothesis testing  Statistical method for sample size determination  Other, specify: *text* |
| **Other** | |
| Any other relevant information | *One answer*  Yes, specify: *text*  No |

| ***Second reviewer*** | |
| --- | --- |
| Reviewer | *One answer*  Carine BELLERA  Cynthia DALLEAU  Derek DINART |
| *Same items as for the first reviewer* | |
