# Supplementary material for: The Growth Modulation Index (GMI) as an Efficacy Outcome in Cancer Clinical Trials: A Scoping Review with Suggested Reporting Guidelines
Source: Curr Oncol Rep. 2025 Mar 29;27(5):516–32. doi: 10.1007/s11912-025-01667-1 (PMC12081581; doi:10.1007/s11912-025-01667-1)
Supplement: Supplementary file 4 — Supplementary file4 (DOCX 59 KB) [file 11912_2025_1667_MOESM4_ESM.docx]

**Table S4** Records briefly mentioning GMI

| **Author(s)** | **Year of publication** | **Title** |
| --- | --- | --- |
| Ashfaq [16] | 2012 | Molecular profiling for personalized cancer care |
| Astsaturov et al. [17] | 2017 | Future Clinical Trials: Genetically Driven Trials |
| Au et al. [18] | 2017 | Personalized and precision medicine: integrating genomics into treatment decisions in gastrointestinal malignancies |
| Basse et al. [20] | 2018 | Exploitation of Precision Medicine Trials Data: Examples of Long Responders From the SHIVA01 Trial |
| Basse et al. [19] | 2018 | Relevance of a molecular tumour board (MTB) for patients' enrolment in clinical trials: experience of the Institut Curie |
| Borad et al. [21] | 2016 | Clinical Implementation of Integrated Genomic Profling in Patients with Advanced Cancers |
| Capper et al. [22] | 2023 | EANO guideline on rational molecular testing of gliomas, glioneuronal, and neuronal tumors in adults for targeted therapy selection |
| Chen et al. [23] | 2019 | Everolimus‐containing therapy vs conventional therapy in the treatment of refractory breast cancer patients with PI3K/AKT/ mTOR mutations: A retrospective study |
| Choucair et al. [24] | 2022 | Liquid Biopsy-based Precision Therapy in Patients with Advanced Solid Tumors: A Real-world Experience from a Community-based Oncology Practice |
| Crimini et al. [25] | 2022 | Challenges and Obstacles in Applying Therapeutical Indications Formulated in Molecular Tumor Boards |
| Czarnecka et al. [26] | 2020 | Molecular Biology of Osteosarcoma |
| De Mattos-Arruda et al. [27] | 2013 | Pilot Studies for Personalized Cancer Medicine: Focusing on the Patient for Treatment Selection |
| Dhani et al. [28] | 2009 | Alternate Endpoints for Screening Phase II Studies |
| Dhir et al. [29] | 2017 | Impact of genomic profiling on the treatment and outcomes of patients with advanced gastrointestinal malignancies |
| Fadoukhair et al. [30] | 2016 | Evaluation of targeted therapies in advanced breast cancer: the need for large-scale molecular screening and transformative clinical trial designs |
| Fountzilas et al. [32] | 2018 | Overview of Precision Oncology Trials: Challenges and Opportunities |
| Fountzilas et al. [31] | 2022 | Clinical trial design in the era of precision medicine |
| Garrido-Laguna et al. [33] | 2011 | The inverted pyramid of biomarker-driven trials |
| Gouda et al. [34] | 2023 | N-of-1 Trials in Cancer Drug Development |
| Hoekstra et al. [35] | 2003 | Clinical trial design for target specific anticancer agents |
| Joffe et al. [37] | 2017 | Clinical Trials in the Genomic Era |
| Kamal et al. [38] | 2018 | Revisited analysis of a SHIVA 01 trial cohort using functional mutational analyses successfully predicted treatment outcome |
| Kim et al. [39] | 2015 | The NEXT-1 (Next generation pErsonalized tX with mulTi-omics and preclinical model) trial: prospective molecular screening trial of metastatic solid cancer patients, a feasibility analysis |
| Kohno [40] | 2018 | Implementation of "clinical sequencing" in cancer genome medicine in Japan |
| Korn et al. [41] | 2013 | Statistical Challenges in the Evaluation of Treatments for Small Patient Populations |
| Kyr et al. [42] | 2021 | N-of-1 Trials in Pediatric Oncology: From a Population-Based Approach to Personalized Medicine-A Review |
| Lanman et al. [43] | 2015 | Analytical and Clinical Validation of a Digital Sequencing Panel for Quantitative, Highly Accurate Evaluation of Cell-Free Circulating Tumor DNA |
| Le Tourneau et al. [44] | 2012 | Tumour growth kinetics assessment: added value to RECIST in cancer patients treated with molecularly targeted agents |
| Le Tourneau et al. [45] | 2014 | The spectrum of clinical trials aiming at personalizing medicine |
| Le Tourneau et al. [46] | 2014 | Randomised proof-of-concept phase II trial comparing targeted therapy based on tumour molecular profiling vs conventional therapy in patients with refractory cancer: results of the feasibility part of the SHIVA trial |
| Le Tourneau et al. [47] | 2016 | Targeted therapies: What have we learned from SHIVA? |
| Menis et al. [48] | 2014 | New clinical research strategies in thoracic oncology: clinical trial design, adaptive, basket and umbrella trials, new end-points and new evaluations of response |
| Meric-Bernstam et al. [49] | 2012 | Overcoming implementation challenges of personalized cancer therapy |
| Mondaca et al. [50] | 2019 | Genomic Characterization of ERBB2-Driven Biliary Cancer and a Case of Response to Ado-Trastuzumab Emtansine |
| Morabito et al. [51] | 2006 | Methodology of clinical trials with new molecular-targeted agents: where do we stand? |
| Morris et al. [52] | 2018 | Performance of next-generation sequencing on small tumor specimens and/or low tumor content samples using a commercially available platform |
| Moscow et al. [53] | 2018 | The evidence framework for precision cancer medicine |
| Naito et al. [36] | 2021 | Clinical practice guidance for next‑generation sequencing in cancer diagnosis and treatment (edition 2.1) |
| Qin et al. [54] | 2019 | Basket trials for intractable cancer |
| Rodon et al. [55] | 2015 | Challenges in initiating and conducting personalized cancer therapy trials: perspectives from WINTHER, a Worldwide Innovative Network (WIN) Consortium trial |
| Russell et al. [56] | 2014 | A practical approach to aid physician interpretation of clinically actionable predictive biomarker results in a multi-platform tumor profiling service |
| Schilsky [57] | 2014 | Implementing personalized cancer care |
| Schork et al. [58] | 2020 | Strategies for Testing Intervention Matching Schemes in Cancer |
| Seymour [59] | 2002 | The Design of Clinical Trials for New Molecularly Targeted Compounds: Progress and New Initiatives |
| Soldatos et al. [60] | 2019 | Precision Oncology-The Quest for Evidence |
| Song et al. [61] | 2023 | Precision Oncology: Evolving Clinical Trials across Tumor Types |
| Stadler et al. [62] | 2000 | Development of target-based antineoplastic agents |
| Stockley et al. [63] | 2016 | Molecular profiling of advanced solid tumors and patient outcomes with genotype-matched clinical trials: the Princess Margaret IMPACT/COMPACT trial |
| Tao et al. [64] | 2018 | Basket Studies: Redefining Clinical Trials in the Era of Genome-Driven Oncology |
| Tayshetye et al. [65] | 2020 | Molecular Profiling of Advanced Malignancies: A Community Oncology Network Experience and Review of Literature |
| Thirasastr et al. [66] | 2022 | Overview of systemic therapy options in liposarcoma, with a focus on the activity of selinexor, a selective inhibitor of nuclear export in dedifferentiated liposarcoma |
| Thomas et al. [67] | 2013 | From targets to targeted therapies and molecular profiling in non-small cell lung carcinoma |
| Thompson et al. [68] | 2019 | Coordinating an Oncology Precision Medicine Clinic Within an Integrated Health System: Lessons Learned in Year One |
| Tsimberidou et al. [69] | 2020 | Review of precision cancer medicine: Evolution of the treatment paradigm |
| Villalona-Calero et al. [70] | 2023 | Integrating Early-Stage Drug Development with Clinical Networks; Challenges and Opportunities: The City of Hope Developing Experience |
| Weiss et al. [71] | 2013 | A Pilot Study Using Next-Generation Sequencing in Advanced Cancers: Feasibility and Challenges |
| Weiss et al. [72] | 2017 | A prospective pilot study of genome-wide exome and transcriptome profiling in patients with small cell lung cancer progressing after first-line therapy |
| Wells et al. [73] | 2014 | Update: The Status of Clinical Trials With Kinase Inhibitors in Thyroid Cancer |
| Weymann et al. [74] | 2021 | Clinical and cost outcomes following genomics-informed treatment for advanced cancers |
| Zhang et al. [75] | 2020 | Molecular Profiling-Based Precision Medicine in Cancer: A Review of Current Evidence and Challenges |
| Zimmer et al. [76] | 2019 | Profiling in Relapsed/Refractory Cancer Patients: A Review Focusing on Latest Profiling Studies |
