# Supplementary material for: The Growth Modulation Index (GMI) as an Efficacy Outcome in Cancer Clinical Trials: A Scoping Review with Suggested Reporting Guidelines
Source: Curr Oncol Rep. 2025 Mar 29;27(5):516–32. doi: 10.1007/s11912-025-01667-1 (PMC12081581; doi:10.1007/s11912-025-01667-1)
Supplement: Supplementary file 5 — Supplementary file5 (DOCX 79 KB) [file 11912_2025_1667_MOESM5_ESM.docx]

**Table S5** Characteristics of documents on interventional studies reporting GMI

| **Author(s)** | **Year of publication** | **Title** | **Design** | **GMI use** |
| --- | --- | --- | --- | --- |
| Adenis et al. [150] | 2013 | A dose-escalating phase I of imatinib mesylate with fixed dose of metronomic cyclophosphamide in targeted solid tumours | Interventional phase I | Secondary/exploratory endpoint |
| Araki et al. [151] | 2016 | Retrospective inter- and intra-patient evaluation of trabectedin after best supportive care for patients with advanced translocation-related sarcoma after failure of standard chemotherapy | Interventional phase II | Secondary/exploratory endpoint |
| Belin et al. [6] | 2017 | Randomized phase II trial comparing molecularly targeted therapy based on tumor molecular profiling versus conventional therapy in patients with refractory cancer: cross-over analysis from the SHIVA trial | Interventional phase II | Secondary/exploratory endpoint |
| Bertucci et al. [152] | 2021 | Prospective high-throughput genome profiling of advanced cancers: results of the PERMED-01 clinical trial | Interventional phase II | Secondary/exploratory endpoint |
| Billon et al. [153] | 2022 | Molecular Profiles of Advanced Urological Cancers in the PERMED-01 Precision Medicine Clinical Trial | Interventional phase II | Secondary/exploratory endpoint |
| Bonetti et al. [154] | 2001 | Use of the ratio of time to progression following first- and second-line therapy to document the activity of the combination of oxaliplatin with 5-fluorouracil in the treatment of colorectal carcinoma | Interventional phase II | Primary endpoint |
| Brodowicz et al. [155] | 2018 | Efficacy and safety of regorafenib compared to placebo and to post-cross-over regorafenib in advanced nonadipocytic soft tissue sarcoma | Interventional phase II | Secondary/exploratory endpoint |
| Buonadonna et al. [156] | 2017 | A noninterventional, multicenter, prospective phase IV study of trabectedin in patients with advanced soft tissue sarcoma | Interventional phase IV | Secondary/exploratory endpoint |
| Byron et al. [157] | 2018 | Prospective Feasibility Trial for Genomics-Informed Treatment in Recurrent and Progressive Glioblastoma | Interventional phase II | Secondary/exploratory endpoint |
| Cardone et al. [158] | 2023 | Regorafenib monotherapy as second-line treatment of patients with RAS-mutant advanced colorectal cancer (STREAM): an academic, multicenter, single-arm, two-stage, phase II study | Interventional phase II | Secondary/exploratory endpoint |
| Cirkel et al. [159] | 2016 | The time to progression ratio: a new individualized volumetric parameter for the early detection of clinical benefit of targeted therapies | Interventional phase II | Primary endpoint |
| Comella et al. [160] | 2002 | Oxaliplatin plus raltitrexed and leucovorin-modulated 5-fluorouracil i.v. bolus: a salvage regimen for colorectal cancer patients | Interventional phase II | Secondary/exploratory endpoint |
| Crisafulli et al. [161] | 2022 | Temozolomide Treatment Alters Mismatch Repair and Boosts Mutational Burden in Tumor and Blood of Colorectal Cancer Patients | Interventional phase II | Secondary/exploratory endpoint |
| De Mattos-Arruda et al. [162] | 2014 | Capturing intra-tumor genetic heterogeneity by de novo mutation profiling of circulating cell-free tumor DNA: a proof-of-principle | Other interventional study | Secondary/exploratory endpoint |
| Debiec-Rychter et al. [163] | 2006 | KIT mutations and dose selection for imatinib in patients with advanced gastrointestinal stromal tumours | Interventional phase III | Secondary/exploratory endpoint |
| Debien et al. [164] | 2023 | Molecular analysis for refractory rare cancers: Sequencing battle continues - learnings for the MOSCATO-01 study | Interventional phase II | Primary endpoint |
| Deeken et al. [165] | 2015 | A Phase 1 Study of Cetuximab and Lapatinib in Patients With Advanced Solid Tumor Malignancies | Interventional phase I | Secondary/exploratory endpoint |
| du Rusquec et al. [166] | 2023 | Evaluation of the relevance of the growth modulation index (GMI) from the FFCD 0307 randomized phase III trial comparing the sequence of two chemotherapeutic regimens | Interventional phase III | Primary endpoint |
| Dumbrava et al. [167] | 2019 | Targeting ERBB2 (HER2) Amplification Identified by Next-Generation Sequencing in Patients With Advanced or Metastatic Solid Tumors Beyond Conventional Indications | Interventional phase II | Secondary/exploratory endpoint |
| Gouda et al. [168] | 2023 | Intrapatient comparative efficacy of selective RET inhibitors using growth modulation index in patients with RET aberrant cancers | Interventional phase II | Primary endpoint |
| Gounder et al. [169] | 2016 | Phase IB Study of Selinexor, a First-in-Class Inhibitor of Nuclear Export, in Patients With Advanced Refractory Bone or Soft Tissue Sarcoma | Interventional phase I | Secondary/exploratory endpoint |
| Heong et al. [170] | 2020 | Whole Exome Sequencing of Multi-Regional Biopsies from Metastatic Lesions to Evaluate Actionable Truncal Mutations Using a Single-Pass Percutaneous Technique | Interventional phase II | Secondary/exploratory endpoint |
| Italiano et al. [171] | 2013 | GDC-0449 in patients with advanced chondrosarcomas: a French Sarcoma Group/US and French National Cancer Institute Single-Arm Phase II Collaborative Study | Interventional phase II | Secondary/exploratory endpoint |
| Italiano et al. [172] | 2020 | Cabozantinib in Advanced Ewing Sarcomas and osteosarcomas: a multicentre, single-arm, phase 2 trial | Interventional phase II | Secondary/exploratory endpoint |
| Jameson et al. [173] | 2014 | A pilot study utilizing multi-omic molecular profiling to find potential targets and select individualized treatments for patients with previously treated metastatic breast cancer | Interventional phase II | Primary endpoint |
| Jiao et al. [174] | 2023 | Targeted therapy for intractable cancer on the basis of molecular profiles: An open-label, phase II basket trial (Long March Pathway) | Interventional phase II | Secondary/exploratory endpoint |
| Kato et al. [175] | 2018 | Rare Tumor Clinic: The University of California San Diego Moores Cancer Center Experience with a Precision Therapy Approach | Interventional phase II | Secondary/exploratory endpoint |
| Kornauth et al. [176] | 2022 | Functional Precision Medicine Provides Clinical Benefit in Advanced Aggressive Hematologic Cancers and Identifies Exceptional Responders | Interventional phase II | Primary endpoint |
| Krebs et al. [8] | 2021 | Intrapatient comparisons of efficacy in a single-arm trial of entrectinib in tumour-agnostic indications | Interventional phase II | Primary endpoint |
| Le Cesne et al. [177] | 2007 | Assessing the clinical impact of trabectedin in patients with leiomyosarcomas or liposarcomas (L-sarcomas) progressing despite prior conventional chemotherapy: clinical benefit rate, growth modulation index and tumor variation as parameters of treatment effect in a randomised international trial of two trabectedin dosing regimens | Interventional phase III | Secondary/exploratory endpoint |
| Lee et al. [178] | 2021 | Phase 2 study of TAS-117, an allosteric akt inhibitor in advanced solid tumors harboring phosphatidylinositol 3-kinase/v-akt murine thymoma viral oncogene homolog gene mutations | Interventional phase II | Secondary/exploratory endpoint |
| Leyvraz et al. [179] | 2022 | Biomarker-driven therapies for metastatic uveal melanoma: A prospective precision oncology feasibility study | Interventional phase II | Secondary/exploratory endpoint |
| Lin et al. [180] | 2021 | Mutational spectrum and precision oncology for biliary tract carcinoma | Interventional phase II | Secondary/exploratory endpoint |
| Mammoliti et al. [181] | 2011 | Two doses of NGR-hTNF in combination with capecitabine plus oxaliplatin in colorectal cancer patients failing standard therapies | Interventional phase I | Secondary/exploratory endpoint |
| Marquina et al. [182] | 2023 | Results of a Phase II Trial Testing the Resensitization With Trabectedin in Platinum-resistant Ovarian Cancer | Interventional phase II | Primary endpoint |
| Massard et al. [183] | 2017 | High-Throughput Genomics and Clinical Outcome in Hard-to-Treat Advanced Cancers: Results of the MOSCATO 01 Trial | Interventional phase II | Primary endpoint |
| Miller et al. [184] | 2022 | Molecular Tumor Board-Assisted Care in an Advanced Cancer Population: Results of a Phase II Clinical Trial | Interventional phase II | Primary endpoint |
| Mir et al. [185] | 2016 | Safety and effi cacy of regorafenib in patients with advanced soft tissue sarcoma (REGOSARC): a randomised, double-blind, placebo-controlled, phase 2 trial | Interventional phase II | Secondary/exploratory endpoint |
| Nagarkar et al. [186] | 2019 | Encyclopedic tumor analysis for guiding treatment of advanced, broadly refractory cancers: results from the RESILIENT trial | Interventional phase II | Secondary/exploratory endpoint |
| Pierobon et al. [187] | 2022 | Multi-omic molecular profiling guide's efficacious treatment selection in refractory metastatic breast cancer: a prospective phase II clinical trial | Interventional phase II | Primary endpoint |
| Prager et al. [188] | 2019 | Results of the extended analysis for cancer treatment (EXACT) trial: a prospective translational study evaluating individualized treatment regimens in oncology | Interventional phase II | Primary endpoint |
| Qin et al. [189] | 2023 | Pan-cancer efficacy and safety of anlotinib plus PD-1 inhibitor in refractory solid tumor: A single-arm, open-label, phase II trial | Interventional phase II | Secondary/exploratory endpoint |
| Radovich et al. [190] | 2016 | Clinical benefit of a precision medicine based approach for guiding treatment of refractory cancers | Interventional phase II | Primary endpoint |
| Réda et al. [191] | 2020 | Implementation and use of whole exome sequencing for metastatic solid cancer | Interventional phase II | Secondary/exploratory endpoint |
| Ree et al. [192] | 2017 | Implementing precision cancer medicine in the public health services of Norway: the diagnostic infrastructure and a cost estimate | Interventional phase II | Primary endpoint |
| Riedl et al. [193] | 2021 | Profiling of circulating tumor DNA and tumor tissue for treatment selection in patients with advanced and refractory carcinoma: a prospective, two-stage phase II Individualized Cancer Treatment trial | Interventional phase II | Primary endpoint |
| Rodon et al. [194] | 2019 | Genomic and transcriptomic profiling expands precision cancer medicine: the WINTHER trial | Interventional phase II | Primary endpoint |
| Seeber et al. [195] | 2016 | Treatment of patients with refractory metastatic cancer according to molecular profiling on tumor tissue in the clinical routine: an interim-analysis of the ONCO-T-PROFILE project | Interventional phase II | Primary endpoint |
| Sicklick et al. [196] | 2019 | Molecular profiling of cancer patients enables personalized combination therapy: the I-PREDICT study | Interventional phase II | Secondary/exploratory endpoint |
| Snijder et al. [197] | 2017 | Image-based ex-vivo drug screening for patients with aggressive haematological malignancies: interim results from a single-arm, open-label, pilot study | Interventional phase II | Primary endpoint |
| Stega et al. [198] | 2020 | A first-in-human study of the novel metabolism-based anti-cancer agent SM-88 in subjects with advanced metastatic cancer | Interventional phase I | Secondary/exploratory endpoint |
| Subbiah et al. [199] | 2023 | Preclinical Characterization and Phase I Trial Results of INBRX-109, A Third-Generation, Recombinant, Humanized, Death Receptor 5 Agonist Antibody, in Chondrosarcoma | Interventional phase I | Secondary/exploratory endpoint |
| Sureda et al. [200] | 2018 | Determining personalized treatment by gene expression profiling in metastatic breast carcinoma patients: a pilot study | Interventional phase II | Secondary/exploratory endpoint |
| Thavaneswaran et al. [201] | 2023 | A signal-seeking Phase 2 study of olaparib and durvalumab in advanced solid cancers with homologous recombination repair gene alterations | Interventional phase II | Secondary/exploratory endpoint |
| Thebault et al. [202] | 2021 | Successive Osteosarcoma Relapses after the First Line O2006/Sarcome-09 Trial: What Can We Learn for Further Phase-II Trials? | Interventional phase III | Secondary/exploratory endpoint |
| Tuxen et al. [203] | 2019 | Copenhagen Prospective Personalized Oncology (CoPPO)-Clinical Utility of Using Molecular Profiling to Select Patients to Phase I Trials | Interventional phase II | Primary endpoint |
| Unseld et al. [204] | 2018 | Feasibility of personalized treatment concepts in gastrointestinal malignancies: Sub-group results of prospective clinical phase II trial EXACT | Interventional phase II | Secondary/exploratory endpoint |
| Verlingue et al. [205] | 2017 | Precision medicine for patients with advanced biliary tract cancers: An effective strategy within the prospective MOSCATO-01 trial | Interventional phase II | Primary endpoint |
| Von Hoff et al. [2] | 2010 | Pilot Study Using Molecular Profiling of Patients' Tumors to Find Potential Targets and Select Treatments for Their Refractory Cancers | Interventional phase II | Primary endpoint |
| Weeber et al. [206] | 2017 | Predicting clinical benefit from everolimus in patients with advanced solid tumors, the CPCT-03 study | Interventional phase II | Secondary/exploratory endpoint |
| Wheler et al. [207] | 2016 | Cancer Therapy Directed by Comprehensive Genomic Profiling: A Single Center Study | Interventional phase II | Secondary/exploratory endpoint |
| Zalcberg et al. [208] | 2005 | Outcome of patients with advanced gastro-intestinal stromal tumours crossing over to a daily imatinib dose of 800 mg after progression on 400 mg | Interventional phase III | Secondary/exploratory endpoint |
