# Supplementary material for: The Growth Modulation Index (GMI) as an Efficacy Outcome in Cancer Clinical Trials: A Scoping Review with Suggested Reporting Guidelines
Source: Curr Oncol Rep. 2025 Mar 29;27(5):516–32. doi: 10.1007/s11912-025-01667-1 (PMC12081581; doi:10.1007/s11912-025-01667-1)
Supplement: Supplementary file 6 — Supplementary file6 (DOCX 82 KB) [file 11912_2025_1667_MOESM6_ESM.docx]

**Table S6** Characteristics of documents on observational studies reporting GMI

| **Author(s)** | **Year of publication** | **Title** | **Design** | **GMI use** |
| --- | --- | --- | --- | --- |
| Ayala de la Peña et al. [78] | 2017 | Evaluation of growth modulation index as a marker of benefit for consecutive lines of treatment for metastatic breast cancer | Observational retrospective study | Primary endpoint |
| Bachet et al. [77] | 2009 | Second- and third-line chemotherapy in patients with metastatic pancreatic adenocarcinoma: Feasibility and potential benefits in a retrospective series of 117 patients | Observational retrospective study | Secondary/exploratory endpoint |
| Bang et al. [79] | 2021 | Clinical outcomes of liposomal irinotecan plus fluorouracil/leucovorin for metastatic pancreatic adenocarcinoma in patients previously treated with conventional irinotecan-containing chemotherapy | Observational retrospective study | Secondary/exploratory endpoint |
| Barata et al. [80] | 2018 | Targeted Next-Generation Sequencing in Men with Metastatic Prostate Cancer: a Pilot Study | Observational retrospective study | Secondary/exploratory endpoint |
| Berclaz et al. [81] | 2023 | Implementing precision oncology for sarcoma patients: the CCC^LMU^ molecular tumor board experience | Observational retrospective study | Secondary/exploratory endpoint |
| Birendra et al. [82] | 2015 | Tumor molecular profiling in the treatment of refractory cancers | Observational retrospective study | Primary endpoint |
| Boilève et al. [83] | 2023 | Molecular profiling and target actionability for precision medicine in neuroendocrine neoplasms: real-world data | Observational retrospective study | Primary endpoint |
| Bruzas et al. [84] | 2021 | Next-Generation Sequencing-Directed Therapy in Patients with Metastatic Breast Cancer in Routine Clinical Practice | Observational retrospective study | Secondary/exploratory endpoint |
| Buechel et al. [85] | 2019 | Investigation of post-immunotherapy response rates utilizing progression free survival ratios in women with gynecologic malignancies | Observational retrospective study | Primary endpoint |
| Bulen et al. [86] | 2023 | Validation of Immunotherapy Response updates Score as Predictive of Pan-solid Tumor Anti-PD-1/PD-L1 Benefit | Observational prospective study | Secondary/exploratory endpoint |
| Chen et al. [87] | 2022 | The Optimal Therapy after Progression on Immune Checkpoint Inhibitors in MSI Metastatic Gastrointestinal Cancer Patients: A Multicenter Retrospective Cohort Study | Observational retrospective study | Secondary/exploratory endpoint |
| Cousin et al. [88] | 2013 | Correlation between overall survival and growth modulation index in pre-treated sarcoma patients: a study from the French Sarcoma Group | Observational retrospective study | Primary endpoint |
| Cousin et al. [89] | 2017 | Clinical impact of extensive molecular profiling in advanced cancer patients | Observational retrospective study | Secondary/exploratory endpoint |
| De Sanctis et al. [90] | 2015 | Efficacy of trabectedin in advanced soft tissue sarcoma: beyond lipo- and leiomyosarcoma | Observational retrospective study | Secondary/exploratory endpoint |
| De Sanctis et al. [92] | 2018 | Predictive Factors of Eribulin Activity in Metastatic Breast Cancer Patients | Observational retrospective study | Secondary/exploratory endpoint |
| Dean et al. [91] | 2016 | Clinical Outcomes of Patients with Rare and Heavily Pretreated Solid Tumors Treated according to the Results of Tumor Molecular Profiling | Observational prospective study | Primary endpoint |
| Dienstmann et al. [93] | 2012 | Molecular profiling of patients with colorectal cancer and matched targeted therapy in phase I clinical trials | Observational retrospective study | Secondary/exploratory endpoint |
| Doleschal et al. [94] | 2023 | Real world evidence reveals improved survival outcomes in biliary tract cancer through molecular matched targeted treatment | Observational retrospective study | Secondary/exploratory endpoint |
| Ducoulombier et al. [95] | 2015 | Growth modulation index (GMI) to assess salvage chemotherapy benefit after FOLFIRINOX progression in metastatic pancreatic adenocarcinoma | Observational retrospective study | Primary endpoint |
| Epelbaum et al. [96] | 2015 | Molecular Profiling-Selected Therapy for Treatment of Advanced Pancreaticobiliary Cancer: A Retrospective Multicenter Study | Observational retrospective study | Primary endpoint |
| Fusco et al. [97] | 2022 | Evaluation of Targeted Next-Generation Sequencing for the Management of Patients Diagnosed with a Cancer of Unknown Primary | Observational retrospective study | Secondary/exploratory endpoint |
| Galland et al. [98] | 2022 | Efficacy of platinum-based chemotherapy in metastatic breast cancer and HRD biomarkers: utility of exome sequencing | Observational retrospective study | Secondary/exploratory endpoint |
| Gallego et al. [99] | 2021 | Bevacizumab in recurrent ovarian cancer: could it be particularly efective in patients with clear cell carcinoma? | Observational retrospective study | Primary endpoint |
| Gambardella et al. [100] | 2021 | Molecular profiling of advanced solid tumours. The impact of experimental molecular-matched therapies on cancer patient outcomes in early-phase trials: the MAST study | Observational retrospective study | Secondary/exploratory endpoint |
| Gebbia et al. [101] | 2007 | Second-line chemotherapy in advanced pancreatic carcinoma: a multicenter survey of the Gruppo Oncologico Italia Meridionale on the activity and safety of the FOLFOX4 regimen in clinical practice | Observational retrospective study | Secondary/exploratory endpoint |
| Giacomini et al. [102] | 2023 | The Molecular Tumor Board of the Regina Elena National Cancer Institute: from accrual to treatment in real-world | Observational prospective study | Secondary/exploratory endpoint |
| Gouton et al. [103] | 2022 | Clinical Impact of High Throughput Sequencing on Liquid Biopsy in Advanced Solid Cancer | Observational retrospective study | Secondary/exploratory endpoint |
| Guiard et al. [104] | 2021 | Impact of previous nivolumab treatment on the response to taxanes in patients with recurrent/metastatic head and neck squamous cell carcinoma | Observational retrospective study | Secondary/exploratory endpoint |
| Guillemois et al. [105] | 2021 | Outcomes in patients treated with taxane regimen after failure of immune checkpoint inhibitors in advanced or metastatic non-small cell lung cancer | Observational retrospective study | Secondary/exploratory endpoint |
| Gutierrez‑Sainz et al. [106] | 2023 | Efficacy of second and third lines of treatment in advanced soft tissue sarcomas: a real‑world study | Observational retrospective study | Primary endpoint |
| Hescot et al. [107] | 2023 | Outcome of adrenocortical carcinoma patients included in early phase clinical trials: Results from the French network ENDOCAN-COMETE | Observational retrospective study | Secondary/exploratory endpoint |
| Hindi et al. [108] | 2020 | Trabectedin Plus Radiotherapy for Advanced Soft-Tissue Sarcoma: Experience in Forty Patients Treated at a Sarcoma Reference Center | Observational retrospective study | Secondary/exploratory endpoint |
| Hoefflin et al. [109] | 2018 | Personalized Clinical Decision Making Through Implementation of a Molecular Tumor Board: A German Single-Center Experience | Observational retrospective study | Secondary/exploratory endpoint |
| Horak et al. [110] | 2021 | Comprehensive Genomic and Transcriptomic Analysis for Guiding Therapeutic Decisions in Patients with Rare Cancers | Observational prospective study | Secondary/exploratory endpoint |
| Ibrahim et al. [111] | 2020 | Comprehensive tumor profiling-guided therapy in rare or refractory solid cancer: A feasibility study in daily clinical practice | Observational prospective study | Secondary/exploratory endpoint |
| Italiano et al. [9] | 2020 | Larotrectinib versus Prior Therapies in Tropomyosin Receptor Kinase Fusion Cancer: An Intra-Patient Comparative Analysis | Observational retrospective study | Primary endpoint |
| Jeong et al. [112] | 2022 | Comparison of the Effectiveness and Clinical Outcome of Everolimus Followed by CDK4/6 Inhibitors with the Opposite Treatment Sequence in Hormone Receptor-Positive, HER2-Negative Metastatic Breast Cancer | Observational retrospective study | Secondary/exploratory endpoint |
| Kankeu Fonkoua et al. [113] | 2017 | Molecular profiling-guided therapy in gastroesophageal carcinoma: A single-institution experience | Observational retrospective study | Primary endpoint |
| Khalifa et al. [114] | 2015 | Efficacy of trabectedin in malignant solitary fibrous tumors: a retrospective analysis from the French Sarcoma Group | Observational retrospective study | Secondary/exploratory endpoint |
| Kobayashi et al. [115] | 2020 | Efficacy and Safety of Trabectedin for Patients With Unresectable and Relapsed Soft-Tissue Sarcoma in Japan: A Japanese Musculoskeletal Oncology Group Study | Observational retrospective study | Secondary/exploratory endpoint |
| Kurz et al. [116] | 2023 | The neuro-oncology magnitude of clinical benefit scale (Neuro-MCBS) as a comprehensive and clinically relevant assessment tool to determine clinical benefit from targeted therapies in CNS tumors | Observational prospective study | Secondary/exploratory endpoint |
| Ky et al. [117] | 2013 | Cisplatin-Modified De Gramont in Second-Line Therapy for Pancreatic Adenocarcinoma | Observational retrospective study | Secondary/exploratory endpoint |
| Lamoureux et al. [118] | 2023 | CNSONTRK study: clinical characteristics and outcome of central nervous system tumors harboring NTRK gene fusions | Observational retrospective study | Primary endpoint |
| Martinez-Trufero et al. [119] | 2017 | Correlation between a new growth modulation index (GMI)-based Geistra score and efficacy outcomes in patients (PTS) with advanced soft tissue sarcomas (ASTS) treated with trabectedin (T): A Spanish group for research on sarcomas (GEIS-38 study) | Observational retrospective study | Primary endpoint |
| Martinez-Trufero et al. [120] | 2021 | A Growth Modulation Index-Based GEISTRA Score as a New Prognostic Tool for Trabectedin Efficacy in Patients with Advanced Soft Tissue Sarcomas: A Spanish Group for Sarcoma Research (GEIS) Retrospective Study | Observational retrospective study | Primary endpoint |
| Merchant et al. [121] | 2008 | A retrospective study of surgery and reirradation for recurrent ependymoma | Observational retrospective study | Secondary/exploratory endpoint |
| Möhrmann et al. [122] | 2022 | Comprehensive genomic and epigenomic analysis in cancer of unknown primary guides molecularly-informed therapies despite heterogeneity | Observational prospective study | Primary endpoint |
| Niger et al. [123] | 2023 | Evaluation of clinical benefit and progression-free survival ratio of targeted treatments in a prospective cohort study of patients with biliary tract cancers | Observational prospective study | Primary endpoint |
| Ning et al. [124] | 2023 | Efficacy and safety of subsequent radiotherapy in patients with advanced-stage hepatocellular carcinoma treated with immune checkpoint inhibitors | Observational retrospective study | Secondary/exploratory endpoint |
| O'Connor et al. [125] | 2018 | Strategy for Assessing New Drug Value in Orphan Diseases: An International Case Match Control Analysis of the PROPEL Study | Observational retrospective study | Secondary/exploratory endpoint |
| Padovan et al. [126] | 2023 | Actionable molecular alterations in newly diagnosed and recurrent IDH1/2 wild-type glioblastoma patients and therapeutic implications: a large mono-institutional experience using extensive next-generation sequencing analysis | Observational retrospective study | Secondary/exploratory endpoint |
| Palmeri et al. [127] | 2022 | Real-world application of tumor mutational burden-high (TMB-high) and microsatellite instability (MSI) confirms their utility as immunotherapy biomarkers | Observational retrospective study | Secondary/exploratory endpoint |
| Peleg Hasson et al. [128] | 2022 | Implementation of Comprehensive Genomic Profiling in Ovarian Cancer Patients: A Retrospective Analysis | Observational retrospective study | Secondary/exploratory endpoint |
| Penel et al. [129] | 2013 | Growth modulation index as metric of clinical benefit assessment among advanced soft tissue sarcoma patients receiving trabectedin as a salvage therapy | Observational retrospective study | Primary endpoint |
| Pernot et al. [130] | 2016 | Reply to the comment on 'Nab-paclitaxel plus gemcitabine for metastatic pancreatic adenocarcinoma after Folfirinox failure: an AGEO prospective multicentre cohort' | Observational prospective study | Primary endpoint |
| Postel-Vinay et al. [131] | 2009 | Clinical benefit in Phase-I trials of novel molecularly targeted agents: does dose matter? | Observational retrospective study | Secondary/exploratory endpoint |
| Purim et al. [132] | 2018 | Biomarker-Driven Therapy in Metastatic Gastric and Esophageal Cancer: Real-Life Clinical Experience | Observational retrospective study | Secondary/exploratory endpoint |
| Quinn et al. [133] | 2021 | Impact of Precision Medicine on Clinical Outcomes: A Single Institution Retrospective Study | Observational retrospective study | Secondary/exploratory endpoint |
| Réda et al. [134] | 2023 | Taxane rechallenge during metastatic disease in HER-2 negative breast cancer patients: Clinical activity, tolerance and survival results | Observational retrospective study | Secondary/exploratory endpoint |
| Renovanz et al. [135] | 2023 | Clinical outcome of biomarker-guided therapies in adult patients with tumors of the nervous system | Observational prospective study | Secondary/exploratory endpoint |
| Repetto et al. [136] | 2023 | Molecular tumour board at European Institute of Oncology: Report of the first three year activity of an Italian precision oncology experience | Observational retrospective study | Secondary/exploratory endpoint |
| Rodriguez-Freixinos et al. [137] | 2019 | Genomic heterogeneity and efficacy of PI3K pathway inhibitors in patients with gynaecological cancer | Observational retrospective study | Secondary/exploratory endpoint |
| Saint-Ghislain et al. [138] | 2022 | MBD4 deficiency is predictive of response to immune checkpoint inhibitors in metastatic uveal melanoma patients | Observational retrospective study | Secondary/exploratory endpoint |
| Salman et al. [139] | 2019 | First-to-second progression-free survival ratio as a predictor for overall survival in ovarian cancer | Observational retrospective study | Primary endpoint |
| Sartore-Bianchi et al. [140] | 2017 | Pooled Analysis of Clinical Outcome of Patients with Chemorefractory Metastatic Colorectal Cancer Treated within Phase I/II Clinical Studies Based on Individual Biomarkers of Susceptibility: A Single-Institution Experience | Observational retrospective study | Secondary/exploratory endpoint |
| Schwaederle et al. [141] | 2016 | Precision Oncology: The UC San Diego Moores Cancer Center PREDICT Experience | Observational retrospective study | Secondary/exploratory endpoint |
| Seeber et al. [7] | 2019 | Treatment According to a Comprehensive Molecular Profiling Can Lead to a Better Outcome in Heavily Pretreated Patients With Metastatic Cancer Data of a Pooled Analysis | Observational retrospective study | Primary endpoint |
| Sultova et al. [142] | 2021 | Implementation of Precision Oncology for Patients with Metastatic Breast Cancer in an Interdisciplinary MTB Setting | Observational prospective study | Secondary/exploratory endpoint |
| Tsang et al. [143] | 2019 | Clinical outcomes after whole-genome sequencing in patients with metastatic non-small-cell lung cancer | Observational retrospective study | Secondary/exploratory endpoint |
| Tsimberidou et al. [144] | 2012 | Personalized Medicine in a Phase I Clinical Trials Program: The MD Anderson Cancer Center Initiative | Observational retrospective study | Secondary/exploratory endpoint |
| van de Kruis et al. [5] | 2022 | The progression-free survival ratio as outcome measure in recurrent ovarian carcinoma patients: Current and future perspectives | Observational retrospective study | Primary endpoint |
| Watson et al. [146] | 2017 | Evaluation of PFS ratio in patients with cancer enrolled in early-phase clinical trials: A single center, retrospective analysis | Observational retrospective study | Primary endpoint |
| Watson et al. [145] | 2018 | Time to progression ratio in cancer patients enrolled in early phase clinical trials: time for new guidelines? | Observational retrospective study | Primary endpoint |
| Wurm et al. [147] | 2023 | Signaling-induced systematic repression of miRNAs uncovers cancer vulnerabilities and targeted therapy sensitivity | Observational retrospective study | Secondary/exploratory endpoint |
| Yoo et al. [148] | 2017 | Efficacy of Chemotherapy in Patients with Unresectable or Metastatic Pancreatic Acinar Cell Carcinoma: Potentially Improved Efficacy with Oxaliplatin-Containing Regimen | Observational retrospective study | Secondary/exploratory endpoint |
| Zhang et al. [149] | 2023 | A Retrospective Analysis of Biliary Tract Cancer Patients Presented to the Molecular Tumor Board at the Comprehensive Cancer Center Munich | Observational retrospective study | Secondary/exploratory endpoint |
