# Supplementary material for: The Growth Modulation Index (GMI) as an Efficacy Outcome in Cancer Clinical Trials: A Scoping Review with Suggested Reporting Guidelines
Source: Curr Oncol Rep. 2025 Mar 29;27(5):516–32. doi: 10.1007/s11912-025-01667-1 (PMC12081581; doi:10.1007/s11912-025-01667-1)
Supplement: Supplementary file 7 — Supplementary file7 (DOCX 25 KB) [file 11912_2025_1667_MOESM7_ESM.docx]

**Table S7** Characteristics of methodological and statistical documents reporting GMI

| **Author(s)** | **Year of publication** | **Title** | **Objective** |
| --- | --- | --- | --- |
| An et al. [221] | 2016 | Time to progression ratio: promising new metric or just another metric? | Discussion/concept |
| Buyse et al. [212] | 2011 | Progression-Free Survival Ratio As End Point for Phase II Trials in Advanced Solid Tumors | Discussion/concept |
| Chen et al. [213] | 2023 | Estimating the Distribution of Ratio of Paired Event Times in Phase II Oncology Trials | Statistical methods for GMI modeling |
| Doroshow [218] | 2010 | Selecting Systemic Cancer Therapy One Patient at a Time: Is There a Role for Molecular Profiling of Individual Patients With Advanced Solid Tumors? | Discussion/concept |
| du Rusquec et al. [214] | 2021 | Drug Development in Tissue-Agnostic Indications | Discussion/concept |
| Freidlin et al. [215] | 2020 | Moving Molecular Profiling to Routine Clinical Practice: A Way Forward? | Discussion/concept |
| Kovalchik et al. [3] | 2011 | Statistical methods for a phase II oncology trial with a growth modulation index (GMI) endpoint | Statistical methods for GMI modeling |
| Le Tourneau et al. [219] | 2012 | Designs and challenges for personalized medicine studies in oncology: focus on the SHIVA trial | Discussion/concept |
| Mick et al. [4] | 2000 | Phase II Clinical Trial Design for Noncytotoxic Anticancer Agents for Which Time to Disease Progression Is the Primary Endpoint | Statistical methods for GMI modeling |
| Mock et al. [220] | 2019 | Community-driven development of a modified progression-free survival ratio for precision oncology | Discussion/concept |
| Paoletti et al. [217] | 2017 | Evaluating Personalized Medicine in Multi-marker Multi-treatment Clinical Trials: Accounting for Heterogeneity | Discussion/concept |
| Samaille et al. [216] | 2022 | Impact of the timing of tumor assessments on median progression-free survival in clinical trials in advanced cancer patients | Discussion/concept |
| Terzer et al. [210] | 2021 | Is the time to progression ratio an appropriate endpoint for clinical trials? A critical examination of current practice and suggestions for a new methodology | Statistical methods for GMI modeling |
| Texier et al. [211] | 2018 | Evaluation of Treatment Effect with Paired Failure Times in a Single-Arm Phase II Trial in Oncology | Statistical methods for GMI modeling |
| Von Hoff et al. [1] | 1998 | There Are No Bad Anticancer Agents, Only Bad Clinical Trial Designs-Twenty-first Richard and Hinda Rosenthal Foundation Award Lecture | Discussion/concept |
| Von Hoff et al. [209] | 2011 | Progression-Free Survival Ratio As End Point for Progression-Free Survival Ratio As End Point for Phase II Trials in Advanced Solid Tumors Reply to M. Buyse et al | Discussion/concept |
| Wu et al. [10] | 2019 | Phase II trial design with growth modulation index as the primary endpoint | Statistical method for sample size determination |
