# Supplementary material for: The Growth Modulation Index (GMI) as an Efficacy Outcome in Cancer Clinical Trials: A Scoping Review with Suggested Reporting Guidelines
Source: Curr Oncol Rep. 2025 Mar 29;27(5):516–32. doi: 10.1007/s11912-025-01667-1 (PMC12081581; doi:10.1007/s11912-025-01667-1)
Supplement: Supplementary file 8 — Supplementary file8 (DOCX 23 KB) [file 11912_2025_1667_MOESM8_ESM.docx]

**Table S8** Characteristics of other types of documents reporting GMI

| **Author(s)** | **Year of publication** | **Title** | **Type of study** |
| --- | --- | --- | --- |
| Bitzer et al. [229] | 2021 | Targeting extracellular and juxtamembrane FGFR2 mutations in chemotherapy-refractory cholangiocarcinoma | Case studies |
| Christofyllakis et al. [224] | 2022 | Cost‑effectiveness of precision cancer medicine‑current challenges in the use of next generation sequencing for comprehensive tumour genomic profiling and the role of clinical utility frameworks (Review) | Review |
| Fraenkel et al. [231] | 2013 | Everolimus therapy for progressive adrenocortical cancer | Case series |
| Heilig et al. [232] | 2021 | Rationale and design of the CRAFT (Continuous ReAssessment with Flexible ExTension in Rare Malignancies) multicenter phase II trial | Protocol |
| Kankeu Fonkoua et al. [222] | 2018 | Molecular Characterization of Gastric Carcinoma: Therapeutic Implications for Biomarkers and Targets | Review |
| Lin et al. [230] | 2019 | The Pros and Cons of Incorporating Transcriptomics in the Age of Precision Oncology | Review |
| Martín-Broto et al. [223] | 2022 | Experience with second-line trabectedin in daily clinical practice: case studies | Case studies |
| Moriwaki et al. [226] | 2016 | Correlations of survival with progression-free survival, response rate, and disease control rate in advanced biliary tract cancer: a meta-analysis of randomised trials of first-line chemotherapy | Meta-analysis |
| Nishikawa et al. [227] | 2018 | A comprehensive review of exceptional responders to anticancer drugs in the biomedical literature | Review |
| Russo et al. [225] | 2023 | Intrathoracic synovial sarcoma with BRAF V600E mutation | Case report |
| Tanigawa et al. [228] | 2017 | Tumors Sharply Increased after Ceasing Pazopanib Therapy for a Patient with Advanced Uterine Leiomyosarcoma: Experience of Tumor Flare | Case report |
